# Potential of frost damage of off-ground foundation stones in Norwegian Stave Churches since 1950 using land surface temperature

**DOI:** 10.1016/j.heliyon.2022.e11591

**Published:** 2022-11-17

**Authors:** Chiara Bertolin, Stefano Cavazzani

**Affiliations:** NTNU - Norwegian University of Science and Technology, Faculty of Engineering, Department of Mechanical and Industrial Engineering, Richard Birkelands vei 2B, 7034, Trondheim, Norway

**Keywords:** Cultural heritage, Stave church, Satellite data, Norway, Freeze-thaw, Foundation stones, Risk, Climate change

## Abstract

The Stave Churches (SCs) are one of the most iconic heritages in Norway, and only 28 medieval SCs have survived till our age in this country. They are built on wood with staves and wooden sleepers standing off the ground on foundation stones which have the primary purpose of guaranteeing structural support, ensuring integrity of the leveled foundation, and avoid ground water or rainwater penetration up to the valuable wooden structures. This paper aims to analyze the risk of Freeze-Thaw (F-T) decay on the foundation stones of the 28 SCs using an algorithm with a single climate input parameter i.e., the land surface temperature, extracted from the Global Land Data Assimilation System GLDAS which integrates satellite- and ground-based observational data products. The novel proposed algorithm estimates a climate-based index of F-T risk on foundation stones starting from the analysis of the variability of a 70-year (i.e., 1950 to 2020) land surface temperature datasets at these 28 locations. The outcome is further used to evaluate the average lifetime (half-life time) of foundation stones as well as the number of interventions necessary to guarantee their structural soundness thus providing a quantitative, predictive, and timely effective information to SC churches managers, and conservators on the climate change effect on foundation stones degradation at support of prioritization of maintenance interventions.

## Highlights

• Use of surface land temperature (ST) from satellite products.
• Creation of an original algorithm to derive a damage function assessing the risk of freeze-thaw cycles impact on off the ground foundation stones.
• Application of the damage function to the still existing 28 Stave churches located in Norway.
• The long-term ST timeseries analysis applied to our algorithm also highlights the impact of climate change at the case studies location.
• Benefit in the use of the novel damage function for prioritizing maintenance intervention, and versatility of applying the risk model to each site.

## 1 Introduction

The off the ground foundation stones (OGFS) of a building have the primary purpose to guarantee structural support to the building, keep out moisture, and insulate the structure from the cold of the ground. This is even most important in case of historic and heritage buildings as the case of the most famous group of heritage buildings in Norway, constituted by Medieval wooden structures (i.e., stavkirker) having stone foundations with off ground parts built between the 11th and the 14th centuries, in Southern and Central Norway, in the regions of Vestland, Vestfold Telemark, Viken, Innlandet, Møre Romsdal, and Trøndelag. Due to the age and significance of this group of heritage buildings - one of them inscribed in the UNESCO World Heritage List (i.e., the Urnes stave church) – a correct survey and effective maintenance of foundation stones is even more important. Risk and first signs of degradation of stone foundations must be monitored and reported to the church manager to plan preventive preservation and extraordinary maintenance or restoration interventions. This is critical not only to ensure the integrity of the leveled foundations over the time for an optimal support of the structure, but even more to make sure ground water or rainwater may not penetrate through the stones of the foundation arriving in contact with the building made of wood. In fact, in such a case water leaking through the OGFS would cause a perfect microenvironment for mold to thrive and grow, or standing water e.g., after heavy or wind-driven rain that could keep wet the stones and/or cause the rot of the wooden structure in contact. Conditions which contribute to keep wet a OGFS are due to the soil around the Stave Churches' (SCs) foundations that being damp and expanding, forces pressure on the foundations, causing water to enter through pre-existing cracks and other openings existing among the stones̀ slabs. Similarly, environmental conditions which mechanically degrade porous stone foundations outdoors are due to the succession of freezing thawing cycles during cold seasons.

In fact, stone material constituted by porous structures ranging from small size to large size [1], during cold seasons, are subjected to numerous freeze-thaw (F-T) transitions from liquid water to ice, which cause in pores fatigue mechanical damage. This is a failure mechanism that involves the cracking of materials and structural components due to cyclic stress generated by the expansion of stone under ice pressure into the pores, resembling cycles of single tensile loads on the surface of the material [2]. As soon as the number of cycles or tensile loads increase, the fatigue damage becomes more likely to occur.

In the knowledge of the authors, few studies exist that evaluate the risk of F-T cycles on OGFS using an algorithm/damage function with a single climate input parameter as the surface temperature. The damage function is a two-time factor exponential decay function aimed at analyzing the long-term impact of freeze-thaw cycles on stone foundations. Similarly, few studies apply such an algorithm to a homogeneous group of heritage buildings spread in different climate zones along a country with the aim of assessing the level of deterioration to prioritize maintenance. This contribution aims to go in this direction i.e., to propose an algorithm - based on the analysis of the surface temperature only - that analyzes the risk of F-T decay on the OGFS of the group of the most outstanding heritage buildings protected in Norway i.e., the 28 existing SCs.

The novel proposed algorithm estimates a climate-based index of F-T risk on OGFS starting from the analysis of the variability of a 70-year (i.e., 1950 to 2020) land surface temperature datasets at these 28 locations (section 2). The outcome of this simple algorithm is further used to evaluate the number of interventions necessary to guarantee structural soundness of the stone foundations (section 3) thus providing a quantitative, predictive, and timely effective information on the climate change effect on their degradation. This information may support SC churches managers, conservators, and most generally stakeholders on prioritization of maintenance interventions on OGFSs. Notwithstanding, this damage function - funded on a single climate variable - is general to be applied in any geographical location where surface temperature datasets are collected, especially in areas where the risk of F-T is expected to increase because of the ongoing climate change as in the case of Central Europe and Scandinavian Countries [3].

### 1.1 State of the art of existing models for risk assessment on stone and built materials by F-T cycles

To control damages caused by wet soil and permanent water infiltration, exterior foundation waterproof repairs on the OGFSs, are required that are long and expensive works. The first step in exterior foundation stones repair is to excavate around the perimeter of the building's foundation. Excavating requires the removal of sections of small portion of landscaping, and walkways around the building. The intervention is therefore (at least at this stage) disruptive. Once the exterior foundation elements and footer are exposed, a drain tile or path is created in the ground around the structure or directly into the stone slabs to be replaced so that the groundwater is directed away from the foundation. When dealing with exterior waterproof repair of historic buildings, this choice is preferable respect to the application of waterproof membrane on the soil as such system do not hold up in the long run and have a visual impact on the significance of the structure. In the case of the whole group of the 28 SCs, during the Stave Church Preservation Programme (SCPP)^1^ [4] - funded by the Norwegian Directorate for Cultural Heritage and lasted from 2001 to 2016 - repair works were conducted to build new foundations and restore from rot damages visible on wooden footer of the buildings. The SCPP carried out maintenance and restoration interventions applying an intervention approach with compatible changes, substantially reversible, or with minimal impact, i.e., implementing the heritage conservation and restoration cautionary approach of “doing as much as necessary but as little as possible” as first introduced in the Burra Charter [5] and in the ICOMOS Principles for the Preservation of Historic Timber Structures [6]. Then as stated in Bakken et al. in 2016 [4], SCPP also followed principles of preserving historic value, original fabric, authenticity, and the intangible knowledge derived by the craftsmen' working techniques, here including their knowledge on detailed condition assessments of the fabric (i.e., level and causes of damage).

Beside the difficulties to define what a minimum intervention is for the built heritage so wide, there is still the need of developing effective tools at support of a prompt prioritization when dedicated budget for in-situ surveys are not available. These tools will help in addressing decisions on where and when implementing interventions, thus solving the ongoing dilemma between minimum and delayed intervention which eventually may lose of effectiveness. In addition, tools that simplify the decision process, help in improving viable conservation practices, in decreasing the rate of necessary maintenance, and in lengthening the period between restoration projects.

Even though the strong efforts made by the local communities over centuries and by the Norwegian Directorate of Cultural heritage in the last decades to preserve and optimally maintains the SCs, they are continuing experiencing the adverse consequences of the local climate in which they are located and even more the ongoing impact of the climate change. Climate change is affecting the cold season in terms of duration and frequency of freezing-thawing events. Although the analysis of the climate variability as temperature (T) trends has a long scientific interest [7], [8], [9], [10]. However, in such context, the further study of its impact on historical heritage materials is a relatively new and stimulating topic. In such framework, the research conducted by the European project Noah's ark project (2004-2007) showed, for the first time using a mapping tool, the anomaly in F-T cycles expected outdoors on stone using the A2 emission scenario derived by the HadRM3 outputs. The damage function implemented by Noah's ark looked at different frost parametrizations, i.e. (1) number of F-T cycles around 0 °C; (2) length of propagated crack (using the formula in Walder and Hallet, [11]); (3) number of rainy days when mean air temperature was >0 °C followed by a day with air mean T < −1 °C representative of the wet-frost days. Finally, the developed damage function by Noah's ark has considered freezing of stone only to occur when T < −3 °C and thawing when T > 1 °C [12]. A step further was done few years later by the EU project climate for culture (CfC, 2009-2015) that used maps to show the expected increase/decrease of risk of mechanical decay by F-T cycles on stones both indoors and outdoors using the same damage function of the Noah's Ark project. This was done evaluating the anomalies in the number of F-T cycles of the far future (FF, 2071-2100) respect to the recent past (RP, 1961-1990) over the whole Europe [3]. All these maps were produced using air temperature projections and following the F-T cycles definition i.e., assessing the times per year (or month, or season) the (air) temperature cycles caused water to pass from the liquid to the solid state (i.e., ice). They agreed in showing – in average - a decrease in the number of F-T cycles in northern European climate.

A Recent dedicated special issue [13] on climate change impact on cultural heritage and historical buildings reports a F-T risk reduction over the FF along the whole Norwegian coast from North to South [14]. Notwithstanding, as already pointed out by Viles [15], van Aarle et al. [16], Sahyoun et al. [17] and by Brimblecombe et al. [12], some areas – especially those at higher latitude in cool continental climate/subarctic climate or those in a Tundra climate zone - are likely to experience a shift towards more mild climate conditions that hover close to zero degrees with a subsequent increase in frost damage potential. This was observed by Loli and Bertolin [14] in the Innlandet (e.g., where the Ringebu SC is located), and Vestfold/Telemark (e.g., where Heddal SC is located) regions in Norway.

Beside the implications on stone buildings and/or OGFSs conservation, these climatic variations are likely to trigger subsidence, thaw-settling, and slope instability effects with a future higher risk of landslides [18], [19], [20], [21], [22], [23].

The study of the sensitivity to frost of stone material (e.g., related to pore characteristics as radius, %; degree of saturation, and water uptake sorption coefficient) is out of the scope of this contribution that is rather focused on the analysis of intensity, rate, and duration of F-T and their modification in the cyclic action over the last seven decades.

As clearly reported by Grossi et al. [19], F-T damage is the result of mechanical stress caused by an increase in water volume when water freezes within the pores. The results of the F-T damage depend on the rock properties, if the rock is soft, with high and low porosity, damage will appear as flacking and granular disintegration at temperatures ranging from −1 °C to −4 °C respectively; while if the rock is hard, the frost damage will happen as fractures and cracks just below 0 °C [24].

Existing models and damage function simulating the decay caused by F-T cycles on stones and/or on building materials adopt single climatological parameter. This is the case of Grossi et al. [19] that use air temperature around 0 °C as simpler notion that freezing and thawing take place below and above this threshold. This same risk threshold is considered in this contribution but using the land surface temperature instead of the air T. Of course, this choice has some limitation as follows: it assumes that liquid water is present so that it can freeze and expand (e.g. source of water may be caused by groundwater or rainwater which saturates the ground); it assumes that the solid-liquid transition occurs at 0 °C, although it is known that this threshold can variate depending on size and distribution of pores in the stone material under examination (e.g. F-T damage is low in materials with coarse pore) and depending on the presence of soluble salts (e.g. F-T damage is low in materials with severe salt contamination, as the case in coastal regions). Although this risk index cannot be strictly used for predicting OGFS damage, it might indicate in which SCs location further investigations are necessary and above all it might assist in decisions about maintenance. In either case, it is useful for relative assessment (i.e., one church respect to the whole group) and when an increasing/decreasing trend in frost damage (or integrity) potential is assessed.

Then other complex climate-based indices that combine several climatological parameters exist. They are all reported in Sahyoun, et al. [25], and were originally modeled by:

– ASHRAE [26] i.e., the semi-empirical Wind-Driven Rain Model that uses rainfall, and wind velocity
– Cornick et al. 2003 [27], i.e., the Moisture Index calculated as a function of hourly values of wetness (WI_h_) and dryness (DI_h_) indices.
– Zhou et al. 2016 [28], i.e., the Climatic Index (CI) calculated as a ratio between annual wetting (i.e., the annual WDR) and drying components (i.e., the annual potential evaporation)
– Salonvaara et al. 2010 [29], i.e., the Severity Index a regression equation developed based on hygrothermal simulations which correlates climatic parameters (e.g., solar radiation, cloud index, wind-driven rain, vapor pressure) with RHT as the performance indicator.

Finally, even more complex functions which quantify the deterioration (integrity) on the built materials are available in literature and are summarized in Table 1.

**Table 1:** Review of existing models and damage functions to simulate F-T decay on stone and other building materials.

Reference	Equation	Eq. N^o^	Parameters involved
Liu et al. 2015, [2]	$D=1-{\left[1-{\left(\frac{N}{N_{d}}\right)}^{1-q}\right]}^{\frac{1}{1+r}}$	(1)	D: damage value; Nd: Max F-T cycle when rock failure; q, r: material parameters by fitting curves to experimental values.
Zhu et al. 2022 [30]	$S_{dr}=1.406e^{\frac{n}{164}}-0.618$	(2)	Sdr: interfacial expansion area ratio of concrete surface; N: number of FT cycles.
Uranjek et al. 2021 [31]	$\mathrm{DI}\left(n\right)=1-\frac{\mu (n)}{\mu_{0}}$	(3)	DI: probabilistic damage index of masonry brick wallet; *μ*: the ductility of damaged (at n F/T cycles) samples; *μ*_0_: the ductility of intact (unconditioned at 0 F/T cycles) samples
Sahyoun et al. 2021 [25]	$\begin{array}{c} \mathrm{MWI}=\sum_{i=1}^{8760}(T_{L}-T_{i})(w_{i}-w_{L})\\ T_{i}<T_{L}\cap \;w_{i}>w_{L} \end{array}$	(4)	MWI: Modified winter index calculates the level of severity where the MC is higher than the critical level *T*_*L*_: critical value of temperature *T*_*i*_:hourly values of temperature at the investigation point *w*_*i*_: hourly values of moisture content at the investigation point *w*_*L*_: critical value of moisture content
Sahyoun et al. 2021 [25]	$\begin{array}{c} {\mathrm{FTDR}}_{\mathrm{index}}=\sum_{\mathrm{cycle}}(S_{\mathrm{Ice},\mathrm{Max}}-S_{\mathrm{Ice},\mathrm{Min}})\\ (S_{\mathrm{Ice},\mathrm{Max}}-S_{\mathrm{Ice},\mathrm{Min}})>0.05 \end{array}$	(5)	FTDR: F-T damage risk index *S*_*Ice*,*Max*_: Max saturation degree of ice content *S*_*Ice*,*Min*_: Min saturation degree of ice content
Ghobadi et al. 2016 [32]; Mutluturk et al. 2004 [33]	*I*_*N*_ = *I*_0_*e*^−*λN*^	(6)	*I*_*N*_: integrity of the tuff after N cycles *I*_0_: original integrity of the tuff *λ*: decay constant N: cycle of F-T
Grossi et al. 2007 [19]	$F=\frac{{\mathrm{DDF}}^{\frac{1}{2}}}{{\mathrm{DDF}}^{\frac{1}{2}}-{\mathrm{DDT}}^{\frac{1}{2}}}$	(7)	F: Surface Frost Index DDF: seasonal degree-days sums above 0 °C DDT: seasonal degree-days sums below 0 °C

– Liu et al. 2015 [2] have formulated the effective frost heaving stress in rocks under the assumptions that pores are spherical, uniformly distributed, and that the water/ice transition process is quasi-static. In their model (eq. (1)), the effective frost heaving stress in stone is equals to the thermal stress minus the ice pressure multiplied by the number of pores. The accumulated damage of rock (D) against F-T is a nonlinear function of frost heaving pressure and the freeze–thaw cycles (N). Thus, in high porous rock, a larger effective frost heaving stress will induce more severe damage.

– Zhu et al. 2022 [30] found a fitting equation (eq. (2)) between the number of FT cycles and the interfacial expansion area ratio. This equation describes the effect in terms of surface roughness of concrete in cold regions (Table 1). In addition, the abrasion rate, depth, and volume decrease with abrasion time in a power law. This information obtained by the fit of the test data allows to provide surface repair measures in time to improve the abrasion resistance of hydraulic concrete.

– Uranjek et al. 2021 [31] proposed a damage model (eq. (3)) based on displacement ductility that can be effective in evaluating the masonry brick degradation under the F/T cycles. The total damage in this model is represented by a value of 1.

– Sahyoun et al. 2021 [25] reported two indices: the modified winter index and the F-T damage risk index, the first (eq. (4)) utilizes hourly values of MC to calculate the level of severity where the MC is higher than the critical level, the freezing is assumed to occur at 0 °C within the material; while the second (eq. (5)) evaluates the accumulation of the difference between the maximum and the minimum saturation degree of ice content in each complete (i.e. process of ice formation and total melting in the porous material) or incomplete (i.e. when freezing activity re-starts prior to the termination of the thawing process) F-T cycle. In fact, following the results of Zhou et al. 2017 [34] incomplete F-T cycles increase the risk of damage therefore the greater the value of the FTDR Index, the greater is the risk of F-T damage

– Mutluturk et al. 2004 [33] and Ghobadi et al. 2016 [32] proposed an exponential function as a decay model to predict the variations of physical and mechanical properties caused by F-T cycles on deteriorated tuff samples. In eq. (6), I_N_ shows the remaining integrity of the material after N cycle, while the decay constant, *λ*, indicates the mean relative integrity loss by the action of each single cycle. These researchers, interestingly, introduced the concept of half-life (i.e., N_1/2_) correlating it with the durability of the tuff material through the equation (8) as follows:

(8) $$N_{\frac{1}{2}}\approx \frac{0.693}{\lambda }.$$

– Grossi et al. 2007 [19] reported a surface frost index (eq. (7)) which may be applied to geographical areas at high latitude. In the function the low index value sets a limit to the existence of permafrost while high values imply continuous permafrost.

When comparing this state of the art it is evident that very few algorithms estimating the potential of frost damage exist with a single climate variable as input [19]. It is also clear that the air temperature is not the optimal climate variable to be selected to evaluate the impact of this potential frost damage on stone materials. To overcome this limitation, several algorithms much more complex have been proposed with climate-based input parameters or with a mix of climate and material response-based parameters. In the first case, they require several climatic data [26], [28] obtained by weather stations or by using hygrothermal building simulations [29], and semiempirical models [27]; in the second case they demand long and costly surveys to gain experimental data to assess the integrity of the material in terms of indexes as damage [2] or integrity [32] value, probabilistic [31], modified winter, surface frost, and F-T [25] damage risk indices or interfacial expansion area ratio [30]. In this study an effort is done to simplify the algorithm without losing in results reliability. This is done selecting a climate variable most sensitive to damage frost potential on OGSF than the air temperature i.e., the land surface temperature.

## 2 Material and methods

### 2.1 The case studies data

**Figure 1:**
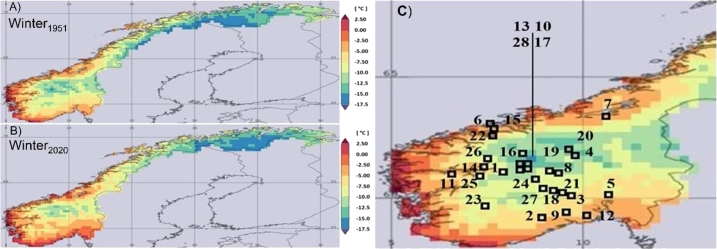
Location of the SCs in the six Norwegian regions: Trøndelag, Møre-Romsdal, Vestland, Vestfold-Telemark, Viken and Innlandet. Comparison between winter (December, January, February) 1951 (A- Top left panel) and 2020 (B- Bottom left panel). C- Stave Churches ID as reported in the first columns in Table 2 (Right panel).

In Norway, the SCs are in the central and southern regions of the country mainly in rural areas, close to waterways, and fjords as visible in Fig. 1c. Norway, over the last 70 years has been impacted by climate change as shown by Fig. 1a and 1b (see section 3 for details). Table 2 reports the complete list of the analyzed churches that represent the case studies data of this contribution. They are ordered alphabetically, identifiable by their latitude, longitude, and by an increasing ID number. In addition, the table reports the köppen climate classes that are: **Dfc** - Humid subpolar climate with short summer and cold winters where permafrost may be common; **ET** - Polar tundra climate; **Dfb** - warm summer humid continental climate with winters that are most cold; and **Dsc** - subarctic climate characterized by long, cold, or very cold winters and short, warm to cool summers. The climate defining criteria of these classes are reported in Table 3.

**Table 2:** ID number, name, municipality, latitude, and longitude of the 28 Stave churches. The köppen climate class, the site type (i.e., C= costal, U= urban, R=rural, M=mountain), their altitude and information on intervention on foundations.

ID, Name	Municipality	Lat [°]	Long [°]	Köppen Class	Site Type	Altitude [m a.m.s.l.]	Intervention on Foundations (Y/NA)
1, Borgund	Lærdal	61.04	7.81	Dfc/ET	C	433	NA
2, Eidsborg	Tokke	59.46	8.02	Dfc/ET	R	551	NA
3, Flesberg	Flesberg	59.86	9.43	ET	R	190	NA
4, Garmo	Lillehammer	61.11	10.47	Dfc	U	240	NA
5, Gol	Oslo	59.90	10.68	Dfb	U	31	NA
6, Grip	Kristiansund	63.21	7.59	Dfc	C	0	NA
7, Haltdalen	Trondheim	63.41	10.35	Dfb	U	152	Y
8, Hedalen	Sør-Aurdal	60.62	9.69	Dfc/ET	R	475	Y
9, Heddal	Notodden	59.57	9.17	Dfc	R	25	NA
10, Hegge	Øystre Slidre	61.15	9.02	Dsc/Dfc	M	659	Y
11, Hopperstad	Vik	61.07	6.57	ET	R	43	NA
12, Høyjord	Andebu	59.36	10.12	Dfb/Dfc	R	111	NA
13, Høre	Vang	61.15	8.80	Dsc/Dfc	R	540	Y
14, Kaupanger	Sogndal	61.18	7.23	ET	R	44	NA
15, Kvernes	Averøy	63.00	7.72	Dfc	C	32	NA
16, Lom	Lom	61.83	8.56	ET	R	363	NA
17, Lomen	Vestre Slidre	61.13	8.92	Dsc/Dfc	R	455	Y
18, Nore	Nore and Uvdal	60.16	9.01	Dfc/ET	R	276	NA
19, Reinli	Sør-Aurdal	60.83	9.49	Dfc	R	544	Y
20, Ringebu	Ringebu	61.50	10.17	Dfc	R	285	Y
21, Rollag	Rollag	60.02	9.27	Dfc/ET	R	225	NA
22, Rødven	Rauma	62.62	7.49	Dsc/Dfc	C	8	NA
23, Røldal	Odda	59.83	6.82	Dfc/ET	R	391	Y
24, Torpo	Ål	60.66	8.70	ET	R	357	NA
25, Undredal	Aurland	60.95	7.10	ET	C	19	Y
26, Urnes	Luster	61.29	7.32	Dsc/ET	C	78	Y
27, Uvdal	Nore and Uvdal	60.26	8.83	ET	R	561	NA
28, Øye	Vang	61.16	8.42	Dsc/Dfc	R	459	NA

**Table 3:** The köppen classes with their climate defining criteria.

Köppen Class	Climate defining criteria
Dfc/ET	Cold/Polar/Tundra (T_hot_>8 & T_cold_<0), cold summer without dry seasons
ET	Polar (T_hot_<10), Tundra (T_hot_>0)
Dfc	Cold, cold summer (T_hot_>10 & T_cold_<0) without dry seasons
Dfb	Cold, warm summer (10<T_hot_<22, T_cold_<0, T_mon10_>4) without dry seasons
Dsc/Dfc	Cold, dry and cold summer (P_sdry_<40 & P_sdry_<P_wwet_/3; T_hot_>10 & T_cold_<0) without dry seasons
Dfb/Dfc	Cold, mild summer (10<T_hot_<22, T_mon10_>4 & T_cold_<0) without dry seasons
Dsc/ET	Cold/Polar (T_cold_<0), cold summer, Tundra (0<T_hot_<10), dry summer ((P_sdry_<40 & P_sdry_<P_wwet_/3)

*Notes:* T_*hot*_ = temperature of the hottest month, T_*cold*_ = temperature of the coldest month, T_*mon10*_ = number of months where the T is >10 °C, P_*sdry*_ = precipitation of the driest month in summer, P_*wwet*_ = precipitation of the wettest month in winter.

Then, Table 2 shows the categorization of the SCs sites in terms of coastal (C), rural (R), urban (U), and Mountain (M) areas. This is done looking at the data collected in Bertolin and Sesana [18] and calculating the distance from the coast (i.e. (C) 3 churches located near a fjord, 4 near the sea with the Grip SC (ID 6) sited on a small island); the high or very low density of dwellings in proximity of the SC i.e., respectively (U) in case of SC relocated in open air museums in urban areas as in Oslo, Trondheim, and Lillehammer or (R) for the remaining 18 churches; and measuring the altitude above the mean sea level. The mountain (M) categorization is provided to Hegge (ID 10) stave church only as it is the only one at an altitude higher than 610 m a.m.s.l. Finally, the last column in Table 2 reports if information on intervention done over the years on the SCs foundations are available (Y) or Not Available (NA). Section 2.2 describes in detail this last information.

### 2.2 Overview of the Stave Churches̀ OGFSs

#### 2.2.1 Existing barriers for Stave Churches̀ OGFSs in-depth investigation

For a stave building, the construction principles are well known and explained in detail in Bakken et al. [4]. It is out of the scope of this contribution, to carry on an in-depth investigation of the conservation status of the entire structures of the churches and of their foundations. Horizontal sections, and above-ground vertical sections of the 28 SC considered in this study were already provided in Bertolin and Sesana 2022, [18] together with their conservation and restoration information.

However in-depth surveys of stone foundations cannot be provided due to the existence of multiple challenges and barriers as (1) long time demand and difficulties in having the simultaneous permission and support by the Directorate of Cultural Heritage and the multiple institutions that own the 28 stave churches (i.e., KA- kirkelig arbeidsgiver- og interesseorganisasjon, Fortidsminneforeningen, and private owners); (2) Lack of a long-term project with a dedicated budget. As an example, the SCPP for which the Norwegian Directorate for Cultural Heritage assumed responsibility, lasted for 15 years and was funded with ca 12.5 million Euro (130 million NOK) from the government funded scheme to protect Norway's historic buildings. This was a unique opportunity which can hardly be repeated in a short time.

To the knowledge of the authors during the SCPP project, investigations of foundations' problems were conducted visually through a careful examination of the beam foundations (i.e., staves) by entering the crawlspace in the church perimeter, examining any lingering wetness, or any signs of a fungi and biological decay on the wooden structure, logs, wooden planks, and joints in contact with the stone constituting the above-ground foundation structure. In case these elements were found wet or rotten, a further investigation was carried on the buried stone foundation structures as those decays could be an indication of possible foundation problems. Similarly, any sign of serious instability of the structure, subsidence, and cracking was also considered an indication of possible stone foundations issues. Nevertheless, despite the availability of budgets, experts, access, and inspection or refurbishment permits, only few above-ground and buried stone foundations (i.e., those recognized as most problematic) were examined in detail through physical exposure.

The physical exposure of the foundations was not done to investigate the type and status of the stone material (i.e., typically by trial pitting, coring, or other techniques), but rather to restore foundations and possibly divert nearby groundwater.

#### 2.2.2 Types of Stave Churches̀ OGFSs and freeze-thaw cycles impact

**Figure 2:**
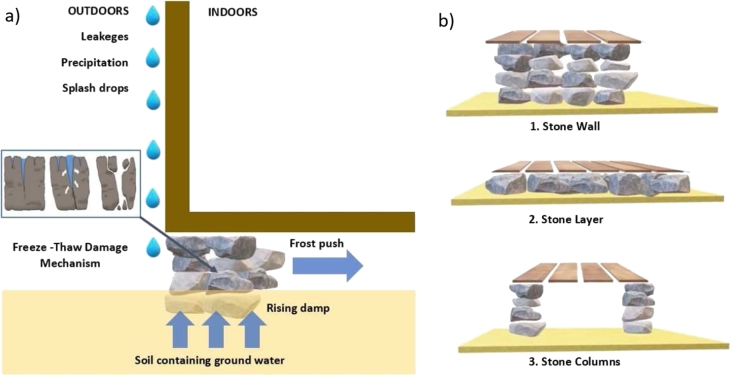
a) Schematic representation of the impact of freeze-thaw cycles on the stone foundations of a SC, with illustrated the sources of liquid water and the three-stage mechanism of fracture (cyan box). b) Scheme of the three types of the stone foundations detectable in the SCs.

This sub-section recaps what is the main F-T damage mechanism which after numerous cycles causes loss of structural integrity to the stone foundations in the SCs (Fig. 2).

Fig. 2a shows how the F-T cycles compromise the stability of the wooden structure by acting on the stones used for the foundations. The risk of decay is enhanced by the availability of liquid water entering the stone pores e.g., through capillary rise (groundwater in very wet soil/sub-soil) and/or directly coming from the roof towards the foundations along the walls of the church (e.g., leakages, precipitation, splash drops) [35]. The decay mechanism can be summarized in a three-steps process (blue box, Fig. 2a). The water absorbed or entered through the stone pores in liquid phase (T > 0 °C, 1^st^ stage) expands during freezing (T < 0 °C, 2^nd^ stage) causing fractures and cracking (3^rd^ stage). The repetition of the decay mechanism through cycles finally breaks the stone (F-T overall damage) [36]. An additional decay mechanism on OGFS is caused by the frost push which tends to displace the dry stones [37].

The 28 SCs have three main types of stone foundations: (1) stone wall, (2) stone layer and (3) stone columns as reported in Fig. 2b. Once the ground is leveled, the dry stones are placed on it following one of the three described configurations. Then, the entire wooden stave structure is built with the main structural pillars (or staves) placed on the stones used for the foundations to avoid contact with the ground. Rising damp in combination with the breaking or displacement of the stones therefore involves an evident compromise of the structural integrity of a SC.

**Figure 3:**
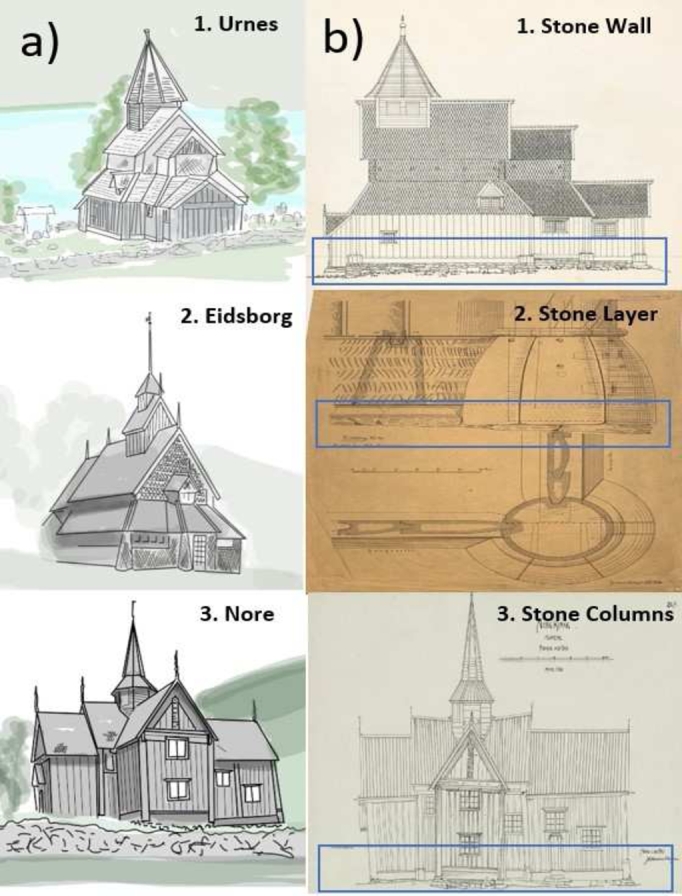
a) 3D reconstruction of three examples of Stave Churches (after Bertolin and Sesana, 2022) in reference to the three types of stone foundations detectable in the stave churches. b) All the historical archive images are available at https://kulturminnebilder.ra.no/fotoweb/ cultural heritage photos from the Norwegian Directorate of Cultural Heritage Archive [38].

Fig. 3 depicts examples of the three original detectable SCs foundations: 1) **Urnes** (ID26, foundations with a stone wall); 2) **Eidsborg** (ID2, foundation with a stone layer); 3) **Nore** (ID 18, foundations with stone columns).

Fig. 3a shows a 3D reconstruction of the entire wooden structure of the church [18], while archival images from the database of the Norwegian Directorate of Cultural Heritage [38], give an indication of the structural load applied to the three types of detected stone foundations (Fig. 3b).

The risk of displacement is most hazardous in compromising the structural integrity of SCs with stone columns foundations (type 3, exemplified in Nore) as visible in Fig. 3b.3, followed by churches with stone wall (type 1, exemplified in Urnes) visible in Fig. 3b.1 and finally by foundations with a layer of stones (type 2, exemplified in Eidsborg) visible in Fig. 3b.2. In an original drawing (20th century, first quarter) of the Nore SC made available by Johannes Kløften, from the Directorate of Cultural Heritage archives (Fig. 3, panel 3b) is clearly visible the loss of structural integrity in form of a noticeable tilt of the entire structure caused by the displacement and breakage of the stone columns used as foundations for the church.

The risk of breakage damages is more hazardous in SCs with stone layer foundations (type 2, e.g., Eidsborg), then in stone column foundations (type 3, e.g., Nore) and finally in churches with stone wall foundations (type 1, e.g., Urnes).

Fig. 3, (panel b.2) shows the vertical (top) and horizontal (bottom) section of the Eidsborg SC above-ground structural configuration. The old illustration (Ca 1890 till 1900) made available by Johan Meyer and preserved in the archive of the Fortidsminneforeningen shows the layer of stones used as a foundation. Any breakage of the stone used to support the main wooden pillar (i.e., the stave) brings the base of the same into semi-contact with the ground, with high risk of rotting and serious structural repercussions.

Concerning the type of stone used in the OGFSs for each church, to the knowledge of the authors documentation reports or catalogues with this information are not available. Most likely this is due to the challenges and barriers already mentioned in section 2.2.1 and in addition to different priorities of research studies that over decades paid more attention to survey: (1) Individual elements (from the foundation stones to cover raft beams and sills) especially in case they are uncommon or abnormal. This information is delivered mainly as drawings; (2) set of existing materials. Often delivered as illustrative information (photos); (3) interventions and changes to trace type and location in the building and/or to trace the age of materials. In such a case available information includes mainly dating and dendrochronology reports; (4) safeguarding measure in case of unexpected and hazardous event. Delivered as management plan e.g., fire safety management.

**Figure 4:**
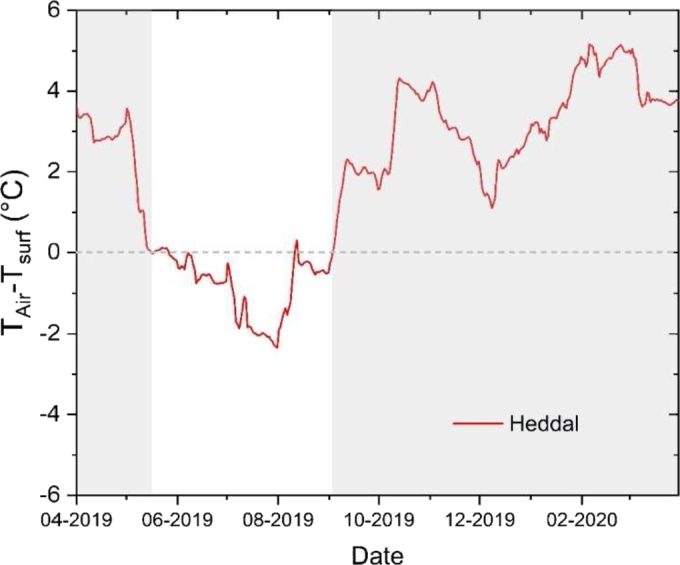
Difference between the outdoor daily mean air temperature and surface temperature (ST) at the Heddal Stave church's location. The Air temperature data were collected outside of the stave church building envelope with a datalogger DL175H1 within the framework of the Symbol project (2018-2022), see Califano et al. 2022a and 2022b [42], [43]. ST data are those analyzed in this contribution. The grey shade highlights the cold season when F-T cycles are possible.

Notwithstanding all the SCs structures were built on stones found in the proximity of the construction area and therefore presumably on stones with local origin. Hansen et al. [39] in Fig. 4, (Map done by P. Storemyr based on data from the Geological Survey of Norway (http://geo.ngu.no/kart/mineralressurser)) report the main types of stones available in the Norwegian territory. In the locations of the 28 SCs they are: Precambrian basement rock (influenced by Caledonian orogeny too), sandstone, magmatic and metamorphic rocks.

#### 2.2.3 Recent stabilization and reconstruction intervention done on the Stave Churches̀ OGFSs

Recently Bertolin and Sesana [18] and Bakken et al. [4] shortly reported as several SCs presented problems in the foundations which were replaced and/or restored during recent decades. This section aims to depict an overview of stabilization and reconstruction interventions done on the stave churches' OGFSs over the last century. The stave church of **Hegge** (ID 10) had restoration works in 1924 conducted by the architect Arnstein Arneberg: on that occasion the rotten foundation stones were removed and a new concrete foundation was cast beneath the staves into solid rock. Later in 2004-2005, during the SCPP, the air ventilation in the foundation was enhanced by deepening the excavation of the underlying terrain. This was done as there was clear evidence of extended damages on the floor and at the base of the wooden structure caused by groundwater seepage.

In **Haltdalen** (ID 7), a SC constituted by wall sleepers on stone foundations, works to build a new stone wall foundation were conducted in 2015 under the framework of the SCPP. In other cases, because of the failure of the foundation that brought to disconnection and poor leveled OGFS, works for straightened the SCs' foundation were conducted two times as in **Lomen** (ID 17) in 1600s and 1800s. This was required because the church was built at the top of a hill at the end of the 12^th^ century, and since then it was subjected to subsidence and slow sliding effects. During its history therefore the church was straightened in more than one occasion up to its reconstruction in 1749. Most recently (2004-2007) during the SCPP program the dry-stone foundation were repaired to uplift the structure which in recent centuries had yielded.

In **Undredal** (ID 25) in 2012 (SCPP program) the church was elevated by about 30 centimeters and a new foundation wall was added to prevent water damage.

In the case of the **Hedalen** church (ID8, mixed type foundation) the foundation wall beneath the church was improved, while in the case of **Reinli** church (ID 19) it was repaired during the SCPP. In both these last two examples detailed archival information with available sketches with numbered stones and/or wall elements were not available, at least not at a scale that allowed to the mason to easily substitute them. During the SCPP, few foundation walls were restored, getting the missing information while building in-situ a mock-up foundation wall that was in this way correctly replicated in the *fabrica* [4]. The SC of **Ringebu** (ID 20) has corner bars that rest on stone slabs, while the wall bars are on stone fill. In the 2010-2015 period (SCPP) at one stave in the church was given a new foundation.

The **Røldal** SC (ID 23) lacks high dry wall foundation. In fact, the construction is almost without foundation (except on the Northern side) as the wall's frames of the *fabrica* are located on a stone level with the ground. **Røldal** was subjected to previous rebuilding and restoration in 1915-1918 and in the last years, in 2015 the low foundation wall on the north side of the church was repaired. The SC of **Høre** (ID 13) had extensive renovations in the 1800s when most of the dry-stone foundation on the southern side was rebuilt. In 2005-2007 period (SCPP) an extensive biological decay was discovered beneath the floor in supporting structures that were replaced; the drainage system was also improved to reduce the risk of water stagnation in the ground in proximity of the church. Finally, the UNESCO WHS of the **Urnes** SC (ID 26) had extensive work of stabilization because of the deterioration of the foundations. In Urnes, the foundations were constituted by simple rock pillars without any stone plinths on the ground to distribute the weight of the structure. Over the centuries this caused structural problems due to the subsidence especially on the north side of the SC. During the SCPP (2007-2011) extensive stabilization works involving the foundations were carried out both encompassing the erection of large stone plinths at the base of the foundations and new dry-stone masonry. This allowed to reassemble the ground floor in connection with the lifting of the church out of the ground.

### 2.3 The surface temperature datasets

Beside the material related to the SCs metadata described in sections 2.1 and 2.2, this work analyzes the potential impact that freezing-thawing cycles may have on the structural integrity of churches̀ OGFS starting from the analysis of the land surface temperature datasets. The surface temperature data are extracted from the Global Land Data Assimilation System GLDAS. In detail, GLDAS integrates satellite- and ground-based observational data products, using advanced land surface modeling and data assimilation techniques, to generate optimal fields of land surface states. NASA GLDAS-2 has three components: GLDAS-2.0, GLDAS-2.1, and GLDAS-2.2. GLDAS-2.0 is forced entirely with the Princeton global meteorological forcing input data [40] and provides a temporally consistent series from 1948 through 2020. GLDAS-2.1 is forced with a combination of model and observation data from 2000 to present. While GLDAS-2.2 product suites use different choices in data assimilation (DA), forcing data, variables, and schemes. Therefore GLDAS-2.0 and -2.1 products are “open-loop” without data assimilation.

The dataset selected in this contribution is the GLDAS-2.0 Catchment Land Surface Model L4 product containing a series of simulated land surface parameters with daily temporal resolution and spatial resolution of 0.25 x 0.25 degree (GLDAS_CLSM025_D). The simulations data are available since January 1, 1948. Simulations use soil moisture and other state fields from the Land Surface Climatology (LSM) as the common GLDAS datasets for land water mask (MOD44W, [41]), the elevation model (GTOPO30), and the default land cover and soils datasets model. It is worthy to mention that the land surface parameters of the Moderate Resolution Imaging Spectrometer on board of Terra (MODIS) are used in the current GLDAS-2.0 and GLDAS-2.1 products too.

The GLDAS-2.0 datasets are analyzed over a 1950-2020 period at the locations of the 28 stave churches as reported in Fig. 1c and Table 2. From now on the GLDAS-2.0 datasets are shortly referred as land surface temperature (ST).

In this study ST over the air temperature has been selected being more representative of the environmental condition at equilibrium with the OGFS. This time series is also the most suitable for the structural integrity impact analysis after the review of the state of the art (section 1.1) and the overview of the F-T damage mechanism described in Section 2.2.2.

An example of the existing difference between air temperature and ST along a calendar year is reported in Fig. 4 for the Heddal (ID9) SC. This yearly reconstruction has been possible for Heddal because it is one of the two SCs where an in-situ microclimate monitoring campaign was recently carried on under the framework of the Symbol- Sustainable Management of Heritage Building in a long-term perspective project (2018-2022) funded by the Norwegian Research Council.

The differences are calculated as differences between the daily mean air temperature – as monitored outside the church using a Testo 175H1 data logger - and the datasets obtained from GLDAS-2.1 over the grid of 0.25° x 0.25°. For details on the monitoring campaign conducted in Heddal (see Califano et al. 2022a and 2022b [42], [43]).

The red line in Fig. 4 clearly highlights positive anomaly during the cold months with the daily mean air temperature always higher than the daily mean ground surface temperature (gray background in the plot) with an average difference equals to 2.4 °C; while a negative anomaly in average equals to −1.1 °C is visible during the warm period from the end of May to the beginning of September (white background in Fig. 4). Therefore, the selection of the ST is more representative of the inertia of the OGFS.

### 2.4 Method for the data processing algorithm development

The methodological approach is divided in two parts, a former focused on the study of the climatological signal embedded in ST datasets; and a latter focused on the development of an algorithm able to discriminate the impact of F-T cycles modifications on OGFS after the calculation of the number of transitions around 0 °C in ST spread. The zero degree at the surface of the material under analysis was used as critical threshold for both freezing and thawing by Sedlbauer and Kunzel [44].

The first part of the methodological steps is as follows:

1. **ST anomaly over the 1950-2020 period.** Two ST anomaly datasets are obtained as daily difference between the single days of each year over the analyzed seven decades (i.e., 1950-2020) and the daily averages of two selected reference thirty-years periods i.e., far past (FP) 1961-1990 and near past (NP)1991-2020 respectively (see later light black and orange lines in Fig. 5). Then the running averages of ST anomalies over a 1-year time window are calculated to better highlight annual inter-variability (Fig. 5, black tick line for the FP 1961-1990 and orange tick line for the RP 1991-2020)

**Figure 5:**
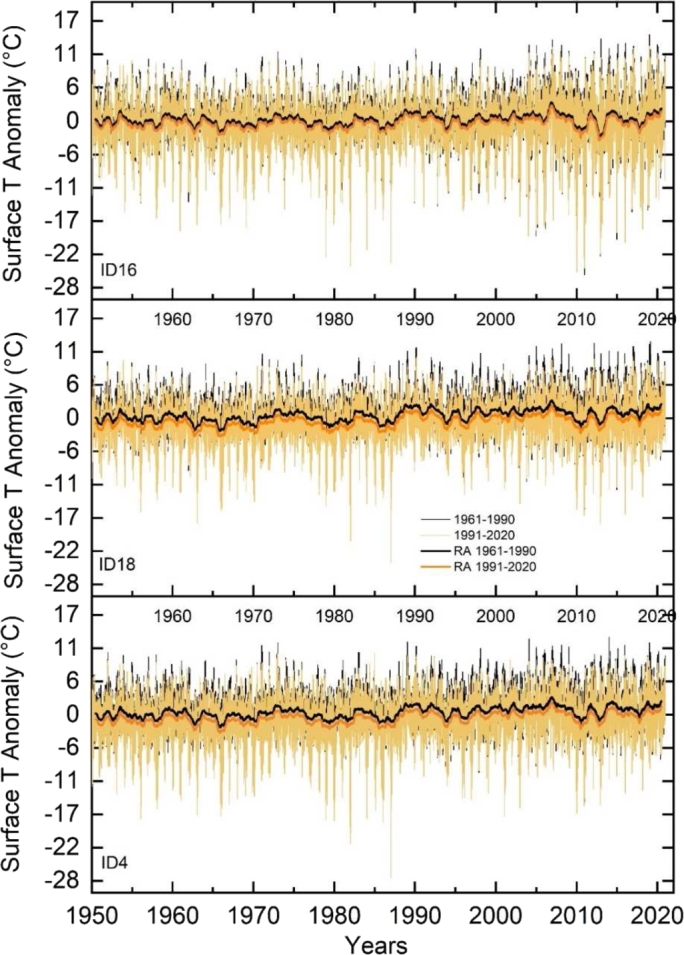
Surface Temperature anomalies respect to the two reference 30-years periods (i.e., FP 1961-1990 and RP 1991-2020) over the 1950-2020 period. The Surface Temperature anomalies are shown for the three risk reference churches: ID 16 high (top panel), ID 18 medium (central panel), ID 4 low risk (bottom panel).
2. **ST ongoing warming trend**. Linear ST data regression allows to estimate the ongoing increase over the 70-years analyzed period.
3. **Fast Fourier Transform (FFT) Analysis of ST.** Beside the slow increasing trend, the major climate change visible on the ST time series is detected by extrapolating the main periodicities over the reference 30-years periods using the Fast Fourier Transform (FFT) as described in detail in the section 3 and reported in Fig. 6.

**Figure 6:**
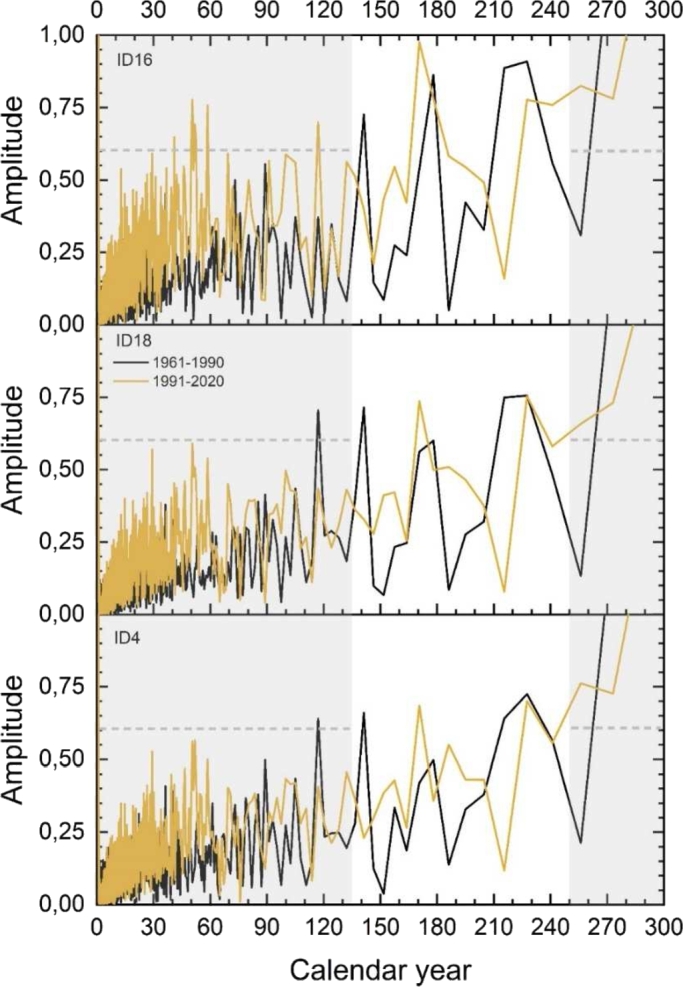
FFT of the two reference 30-years (1961-1990 and 1991-2020) for the three risk reference churches: ID 16 high (top panel), ID 18 medium (central panel), ID 4 low risk (bottom panel). The gray area highlights the winter season. The dashed line delineates a threshold that shows how in a high-risk church the width and number of peaks increases compared to a low-risk church.

While the second part is reported below:

1. **Calculation of the standard deviation** (SD) of the ST over the time intervals highlighted by the FFT main periodicity i.e., 3 days (SD_3DD_, Fig. 7 – left panel) and 7 days (SD_7DD_).

**Figure 7:**
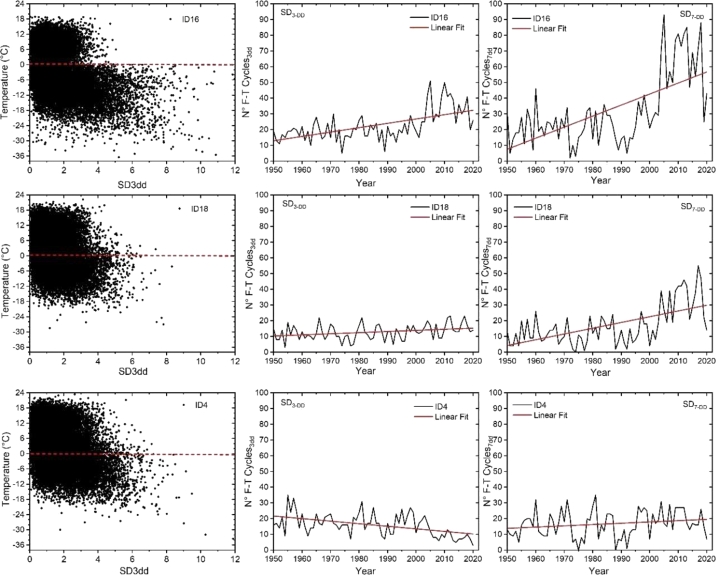
Comparison between the three risk reference churches: ID 16 high risk (top), ID 18 medium risk (central), ID 4 low risk (bottom). The left panels show the 3-days ST standard deviations with the respective trends in the number of critical events around 0 °C from 1950 to 2020 (central panels). The right panels show the 7-days number of events trend around 0 °C from 1950 to 2020, and the linear regression used to evaluate the risk index.
2. **Count of the yearly number of events** across the 0 °C ST threshold by the SD_3DD_ and SD_7DD_ variability. This is a measure of the increase, year-by-year, in number of freezing-thawing dispersion phenomena (Fig. 7, center panel for the SD_3DD_ of the ST; right panel for the SD_7DD_ of the ST). The SD_7DD_ value provides an indication of the duration of the freezing and thawing weeks. The midweek SD_3DD_ value analyzes how this freeze thaw phenomenon occurs. In case of high SD_7DD_, freeze-thaw cycles have occurred during the week. In case of both high SD_7DD_ and SD_3DD_, F-T cycles are repeated midweek, subjecting the structure to continuous stress. On the contrary, if SD_7DD_ is high, but SD_3DD_ is low, it implies that the number of freeze-thaw cycles per week are less, and the structure has not undergone continuous stress.
3. **Calculation of the linear regression**. The angular coefficient of the linear regression calculated on the number of F-T cycles with large measure of variability is evaluated to show the exacerbation of climate change impact over the 1951-2020 period.
4. **Estimation of a novel decay function (LT (t)).** To model the lifetime of the SCs OGFSs a new decay function is proposed. The exponential coefficient of this decay function is constituted by two factors, each one related to the change in periodicity of the F-T dispersion as highlighted by the FFT analysis i.e., 3 days (first factor, N_D1_) and 7 days (second factor, N_D2_). The algorithm employed to detect damage is built based on all the above-described methodological steps as the exponential coefficient contains, the angular coefficients representative of the long-term trends of F-T cycles (i.e., m_1_ and m_2_, Fig. 8). The lifetime function LT(t) is finally calculated for all the 28 SCs locations (Table 4).

**Figure 8:**
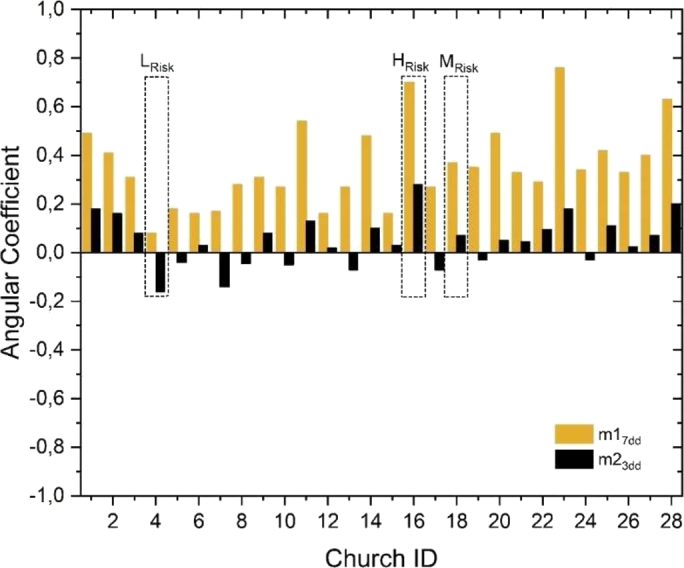
Histogram of the SD_7DD_ and SD_3DD_ angular coefficients of the 28 SCs. The low-risk church ID4, the medium-risk church ID18 and the high-risk church ID16 are highlighted.

**Table 4:** Headers: ID of the SCs. Lines 2 and 3: angular coefficients at 7 days (m1) and 3 days (m2). Coefficients in green do not present risks in relation to *LT*(*t*)=*e*^−*α***⋅***t*^. Line 4: decay constant. Line 5: OGFS half-life. Line 6: Number of restorations needed over the 200-year time horizon.

	1	2	3	4	5	6	7	8	9	10	11	12	13	14
**m**_1_	**0.49**	**0.41**	0.31	*0.04*	*0.18*	0.16	*0.17*	*0.28*	0.31	*0.27*	**0.54**	0.16	*0.27*	**0.48**
**m**_2_	**0.18**	**0.16**	0.08	*0.16*	*0.04*	0.03	*0.14*	*0.05*	0.08	*0.05*	**0.13**	0.02	*0.07*	**0.10**
***α***	**0.0074**	**0.0055**	0.0021	–	–	0.0004	–	–	0.0021	–	**0.0059**	0.0003	–	**0.0040**
**t**_50%_	**94**	**127**	335	–	–	1733	–	–	335	–	**118**	2599	–	**173**
**N**	**2.1**	**1.6**	0.6	–	–	0.1	–	–	0.6	–	**1.7**	0.1	–	**1.2**

	15	16	17	18	19	20	21	22	23	24	25	26	27	28

**m**_1_	0.16	**0.70**	*0.27*	0.37	*0.35*	**0.49**	0.33	0.29	**0.76**	*0.34*	**0.42**	0.33	**0.40**	**0.63**
**m**_2_	0.03	**0.28**	*0.07*	0.07	*0.03*	**0.05**	0.05	0.10	**0.18**	*0.03*	**0.11**	0.03	**0.07**	**0.20**
***α***	0.0004	**0.0163**	–	0.0022	–	**0.0020**	0.0014	0.0024	**0.0114**	–	**0.0039**	0.0008	**0.0023**	**0.0105**
**t**_50%_	1733	**42**	–	321	–	**340**	504	287	**61**	–	**180**	840	**297**	**66**
**N**	0.1	**4.7**	–	0.6	–	**0.6**	0.4	0.7	**3.3**	–	**1.1**	0.2	**0.7**	**3.0**
5. **Identification of the 10 most in danger SCs OGFSs and of the number of interventions**. Once fixed 200 years as the time horizon for the standard duration of half-life time (t_50%_) of a stave church OGFS; Fig. 9 and the LT(t) function (i.e., equation (9)) may be used as tool to set the need of maintenance actions. Here, the hypothesis is that the full life durability of structural supporting OGFSs under acceptable/standard decay processes caused by F-T cycles, cracking, flacking, water infiltration, and leakage is of circa 400 years. Therefore, looking at the time in which the LT curve of each specific SC crosses the t_50%_ threshold, it is possible to estimate the required number of interventions over a 200-yr time horizon.

**Figure 9:**
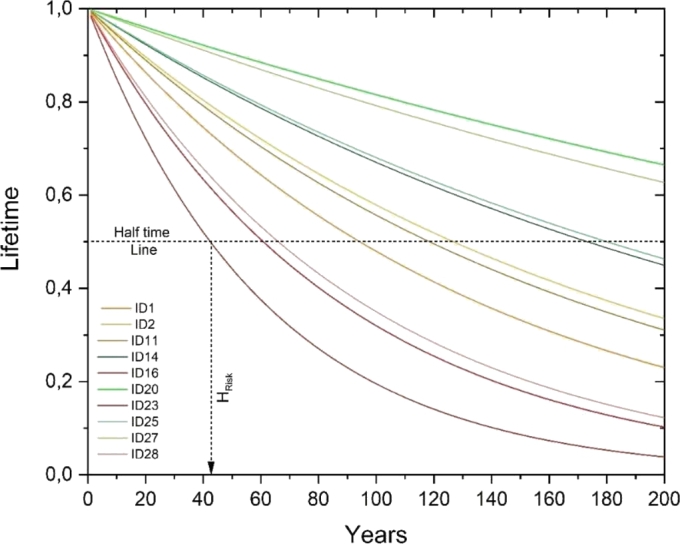
Lifetime function of the 10 churches at greatest risk with their ID. Figure shows a simulation of the conservative horizon over a 200-year horizon assuming similar climatologic conditions as those analyzed over the 1950-2020.

SCs that need more than one restoration intervention on their foundation are here considered at **High risk**; SCs that require a single intervention over 200-250 years are considered with normal/standard durability basements (**Medium Risk**); while those churches demanding a restoration of foundation once every 400 years are regarded as no risky for the effect of F-T cycles (**Low risk**). These hypotheses of course are reliable if the foundations are waterproof with a proven subfloor system that can discharge groundwater aways from the wooden structures. Table 4 reports the exponential coefficient, the t_50%,_ and the number of interventions (NR) over a 200-yr horizon for the whole group of the 28 stave churches. Exemplificative churches with Low (Garmo, ID4), Medium (Nore, ID18) and High risk (Lom, ID16) categorization are used to present the results in Section 3.

## 3 Results and discussion

Fig. 1 above shows the comparison between the mean winter (i.e., December, January, and February) ST in 1951 and 2021 (Fig. 1a and 1b). The map in Fig. 1c also shows the spatial resolution of the 0.25° × 0.25° matrices used for the 28 churches with their ID number as reported in Table 2. In this contribution, to analyze the impact of freezing-thawing cycles on OGFS a code was used to calculate the number of high temperature dispersion events near 0 °C harmful to these 28 locations.

The analysis carried out and the type of code used are combined with this spatial resolution, in addition using a signal averaging technique over these matrices, the strength of a signal relative to noise (SNR) is increased thus improving the data stability at site locations adjacent to SCs.

The maps in Fig. 1 clearly show mild mean winter temperature along the Norwegian coasts (i.e., ST>-5 °C) and simultaneously a ST spatial decrease in the region $\left[\begin{array}{ccc} \; & 62.5^{\circ }\; & \\ 7.5^{\circ }\; & \; & 11.5^{\circ }\\ \; & 60^{\circ }\; & \end{array}\right]$ with values approaching mean winter ST <-10 °C in the inner land. The comparison of Fig. 1a and 1b clearly shows the impact of climate change over the last 70 years.

Looking at the climate conditions in winter 2020 respect to those in winter 1951, a local temperature increase is accentuated mainly in the inland with a spatial reduction of isoline-surfaces with mean winter ST < −10 °C. This warming is higher in the inter-seasons period i.e., autumn-winter and winter-spring and as an overall effect it manifests itself with an increasing in the F-T cycles. Icy phenomena also increased, causing the annual dispersion of temperatures to rise. This means colder, shorter winters accompanied by warmer, longer summers. The stress load to which the OGFSs are subjected is therefore greater today respect to 1951. While the trend in daily to annual ST shows a limited increase (see Fig. 5), the number of variations of hot days, year by year, has increased markedly.

Fig. 5 shows the anomalies (i.e., yearly ST in each year minus yearly ST calculated over the two reference 30-years periods i.e., FP and RP) for the three exemplificative churches used to describe high, medium, and low risk. The bottom panel reports the ST anomaly at the location of a church suffering low climate change impact (ID 4, Garmo), the middle panel the ST anomaly at a location with medium impact (ID 18, Nore), while the top panel at a location with high impact (ID 16, Lom). In the plots the 30 days running average over the FP and the RP shows the same variability with a small vertical downward shift of 0.5 °C as average value for the RP. As expected, this demonstrates as the anomalies are irrespective to the selected 30-years reference period. In the high-risk location, the greatest anomaly is evident over the past 15 years in terms of (1) wider anomaly spread, (2) deeper and more frequent negative anomaly peaks and (3) floating yearly average with higher anomalies peaks in module. It also shows a long-term periodicity in addition to the annual cycle. Climatologically, this implies a recent increase in extreme phenomena and a decrease in the duration of winters.

The Fourier's analysis, that consists in the possibility of representing complicated periodic structures using combinations of simple periodic functions, has been implemented to better analyze the periodicity of the ST timeseries. The Fourier's analysis allows switching between the time and frequency domain for a physical phenomenon. A function can be described by its value f(t) or by its amplitude F(w) with frequency w. A Fourier transform (FT) converts f(t) into F(w) and vice versa.

However, since data are discrete, in the analysis we used the discrete Fourier transform (DFT) to convert a real or complex vector into another with components that are a combination of periodic functions. The DFT is the equivalent of the continuous FT for signals known only at instants separated by sample times (e.g., a finite sequence of data).

Specifically, the use of the Fast Fourier Transform (FFT) extrapolates the main periodicities (e.g., short term seasonal periodicity) of a series of data. These periods can be used in climate and forecast models (Cavazzani et al. 2017, 2019, and 2020 [45], [46], [47]). The FFT is an algorithm for the fast calculation of the DFT. The strategy underlying FFT algorithms is to change the calculation of a DFT of length N to the calculation of a lower DFT length. In radix-2 FFT algorithms, a DFT of length N = 2^n^ is reduced to the calculation of a length 2 DFT [48].

Fig. 6 reports the result for the three risk reference churches. The analysis refers to the two thirty-years of reference: 1961-1990 (FP, black line) and 1991-2020 (RP, yellow line) and shows the change in the length of the seasons and the increase in dispersion in the first months of the year. In the church with ID16, the impact of climate change causes the greatest differences between the two signals.

The gray area of the graphs indicates the cold period, and it shows how for a high-risk church one of the peaks related to the winter-spring change of season is brought forward to the day 130 in the RP (yellow line) compared to its previous occurrence at day 140 (mid-April) in the FP 1961-1990 (black line). Fig. 6 also shows the increase in signal amplitude during the cold months (January-February-March). In particular, the peaks of February and March have variations around 0 °C that infer more stress on the stone structures. Similarly, the white area shows a 210-day peak (late July) in the FP that is delayed up to day 230. This can be interpreted as an extension of the warm period by approximately 30 days (percentile analysis).

Interestingly, in the other two churches (ID18 and ID4) these peaks are not displaced, but simply they are damped, indicating less marked changes in temperature (milder winters).

In the recent past (1991-2020), the analysis of the church at high risk shows other noticeable changes: (1) three distinct peaks that exceed a horizontal threshold (dotted line in Fig. 6) during the first months of the year (thirty years 1991-2020) confirming greater repeated thermal excursions, harmful to the structural integrity of the OGFSs; (2) a stable (horizontal) signal (from day 230 to day 270) with small fluctuations indicating a poorly defined autumn-winter change of season that over the decades has been postponed (late winter).

In consideration of these results, Fig. 7 reports the three basis steps in the algorithm adopted to develop the proposed damage function that are:

(1) the analysis of the weekly (7 days) and mid-week (3 days) standard deviation (SD) to extrapolate a reference threshold around 0 °C.
(2) the representation of the trend of the number of SD_7DD_ and SD_3DD_ cycles above this threshold (step 1 above) for the 28 SCs over the last 70 years.
(3) Computation of the linear regressions' angular coefficient of the number of cycles at SD_7DD_ and SD_3DD_.

Fig. 7 shows the first two steps for the three risk reference churches.

The high-risk church (ID16, top plots) has a very pronounced 0 °C threshold, accompanied by a sharply increasing number of SD_3DD_ and SD_7DD_ cycles over the past 20 years with their linear regression depicted as red line. This means that in this area, there is an increasing number of weeks in which the temperature repeatedly goes from below to above 0 °C also confirmed in the number of midweek events.

The low-risk church (ID4, bottom plots) at the opposite shows a relatively constant SD_7DD_ cycles and decreasing SD_3DD_ cycles. The use of two-time intervals allows a verification of the results and describes the seasonal changes in terms of weekly and midweek ST variations around 0 °C.

While Fig. 8 shows the third and last step i.e., the results of the angular coefficients calculated for the 28 SCs with highlighted the three risk reference churches. The bars in yellow report the coefficients calculated for the SD_7DD_ while the bars in black those for the SD_3DD_. The comparison shows the churches more subject to the impact of these transition periods, allowing for an immediate risk assessment. The calculation of the two intervals (7 days and 3 days) guarantees a self-consistent verification of the result. The analysis shows – for several SCs - an increase in the weekly transition periods but a decrease in the midweek ones. In the case of negative angular coefficients (m<20), the risk fraction becomes very low. This means that the stone surface is not subjected to continuous transitions during the week, but the dispersion occurs on weekly intervals, thus decreasing the load on the foundation stones. Furthermore, in the cases of SCs with negative m_2_, the m_1_ are generally also low thus further reducing the overall risk.

### 3.1 Novel damage function and overview of the potential risk of F-T cycles on OGFSs of the 28 stave churches

As mentioned also in Bertolin and Sesana [18] related to the DRR policy inherent to SCs, policy makers, stakeholders, and heritage institutions managers have an increasing need of raising awareness not only towards risk of natural hazards at the location of the SC, but also towards the proper capacity of recognizing small and continuous climate change impacts which may affect their capacity to maintain optimal conditions of building and OGFSs. With the objective of preserving the whole group of SCs affected by climate change, a novel damage function i.e., the lifetime function LT (t) (see Equation (9) below) that estimates the lifetime of foundation stones is presented. It assesses the rate of integrity loss due to the potential weathering effect of freezing-thawing cycles proportionally to the rock integrity at the beginning of the life. It is of support to stakeholders in conducting a F-T risk assessment and in prioritizing OGFSs maintenance, overcoming the existing barriers of making research more open, understandable, and usable especially in case of lack of dedicated budget for conducting in-situ survey to assess OGFS integrity.

In detail, the ST time series provides the algorithm with data of input to enumerate the transition of F-T events after having evaluated the ST standard deviation in weekly (SD_7DD_) and midweek (SD_3DD_) intervals. By enumerating these events (N_D1_ and N_D2_), the increase (linear) trend over the 1950-2020 period becomes evident. The angular coefficients (m_1_ and m_2_) of the linear regressions in the two SD_7DD_ and SD_3DD_ datasets become the parameters in the proposed lifetime function LT (t) that has the time as independent variable. The novel algorithm is as follows:

(9) $$\mathrm{LT}\left(t\right)=e^{-\left[\left(\frac{m_{1}}{N_{D_{1}}-1}\right)\cdot \left(\frac{m_{2}}{N_{D_{2}}-1}\right)\right]\cdot t}$$

(10) $$\mathrm{LT}(t)=e^{-\alpha \cdot t}$$

(11) $$t_{50\text{\%}}=\frac{-\ln\left(0.5\right)}{\alpha }$$

(12) $$N=\frac{200}{t_{50\text{\%}}}.$$

The coefficient *α*, that describes all the parameters into the square brackets (equation (10)), is the decay constant as it expresses the disintegration rate of stone; while t_50%_ (equation (11)) is the half-life i.e., the time expressed in number of years after which there is a degradation of the structural integrity of a material or component of 50%. In equation (12), N is the number of restoration interventions on the OGFSs estimated considering a fixed horizon of 200 years. Table 4 reports the calculations done to apply this novel algorithm to the locations of the 28 SCs. It shows results for the angular coefficients at 7 days (m_1_) and 3 days (m_2_), the decay constant *α*, the half-life, and the number of restorations needed over a 200-year time horizon (N). The symbol “-” refers to churches with negative m_2_ and low risk, for which the calculation of *α*, t_50%_ and N is not significant, and the durability of OGFSs exceeds the life horizon of 200-year. In particular, this is the case of the Garmo stave church (ID4 in Figure 8, Figure 9) for which the LT (t) equation becomes not significant. While the values in bold are those of the 10 churches most at risk that are referred to Fig. 9.

When the lifetime is reduced, the stress to which the foundation stones are subjected is higher.

Fig. 9 compares the LT (t) over the common 200-year time horizon for the 10 churches with reduced lifetime. The stave churches with higher risk (descending order) have ID 16, 23, 28, 1, 11, 2, 14, 25, 27, 20 respectively.

When an extraordinary maintenance or a restoration intervention is just done, the OGFSs show a maximum integrity value i.e., LT (0) =1. In the lifetime simulation carried out using the novel LT(t) at the 28 SCs locations, the results of which are shown in Table 4 and in Fig. 8, all the churches have therefore the same starting lifetime (i.e., t=00) and the same initial zero integrity level (i.e., LT (0)=1). As soon as the life of the OGFSs of each SC progresses - over a common horizon of 200-year – its decay depends on the F-T cycles occurring at its specific location. This is due to the different decay constant that modifies the durability.

In Fig. 9, the dotted horizontal line indicates the half-life corresponding to an indicative 50% degradation of the OGFSs. The counterimage of this point (x-axis) indicates the time interval in which, a restoration intervention is recommended. This number corresponds to the last line (N) in Table 4 that for the 10 churches most at risk of consequences from F-T cycles ranges between 0,6 and 4,7 i.e., a recommendation of intervention every 360 to 42 years, respectively. Fig. 9 reports in red and dark orange shades the SCs with OGFS having half-life < 100 years (4 over 10 SCs). They are the churches of Lom (ID16), Røldal (ID23), Øye (ID28), and Borgund (ID1) respectively. These churches are located in polar and tundra climate zone (ET, Lom SC) with average temperature of the hottest month ranging between 0 °C and 10 °C; in zones with cold polar tundra climate having average temperature of the coldest month below zero (Dfc/ET, Røldal and Borgund SCs); and in zones with cold climate and dry and cold summer i.e., with average temperature of the coldest month below zero and with an average temperature of the hottest month >10 °C with few precipitation (driest month in summer <40 millimeters), (Dsc/Dfc, Øye SC).

In literature, there are no clear thresholds in number of F-T cycles above which severe damage on building materials is observed. One of the few works reporting such thresholds is that of Ruedrich et al. [49] that correlates F-T cycles number with degradation occurrence on sensitive samples constituted of limestone, granite, and tuff. They reported as after 1400 F-T cycles their samples in limestone showed large cracks appearance and a reduced loss in weight; samples in granite showed sugar like crumbling at the surface; while samples in tuff collapsed after 1055 F-T cycles with a strong loss of material. In their study (except for very soft stone) a 1400 F-T cycles threshold was representative to visually discriminate the integrity of several types of rocks (i.e., macro damages appearance). In this study, the proposed algorithm detects the weeks in which there are freeze-thaw cycles thanks to the SD_7DD_ analysis in relation to the 0 °C threshold. The SD_3DD_ assesses the criticality for the foundation stones in these weeks, providing indications on the midweek freeze-thaw cycles. Then the half-life of a selected time horizon (here, 200-years) helps in categorizing F-T damage potential on the OGFS. Based on the total number of F-T cycles we detected, with the algorithm over the 1950-2020 period, the discrimination threshold for high damage potential agrees with the finding of Ruedrich et al. [49]. In fact, the SC at highest risk, Lom (ID16), was subjected over 70 years to an increase in both the 3-day (total 1606 events from 1950 to 2020) and 7-day freeze-thaw events (total 2282 events from 1950 to 2020).

At the opposite, the stave church at lowest risk i.e., the church of Garmo (ID4, not visible in Fig. 9) was subjected to an almost constant number of 7-day freeze-thaw events (total 1182 events from 1950 to 2020) and a decrease in 3-day freeze-thaw events (total 1132 events from 1950 to 2020). In this case, reducing vulnerability, climate change has a positive effect on OGFS durability (Fig. 8).

Notwithstanding an evaluation of number of F-T cycles in relation to SD_7DD_ and SD_3DD_ at higher temporal resolution (i.e., hourly, or daily) will be the subject of future work. The further objective will be the analysis of hourly temperature trend using in situ monitoring devices i.e., surface temperature sensors located on stone surface and air temperature sensors to compare the values of the freeze-thaw events detected by the algorithm and the exact number of freeze-thaw cycles measured in situ by the sensors. This empirical calibration will implement the proposed LT(t) damage function.

At this stage of the research, the verification in the use of the LT(t) can only be partial looking at the data collected in Table 2 related to restoration works on the OGFSs of the SCs because of the lack of long-term, homogeneous historical documentary sources. From the comparison between the calculated number of F-T cycles and the past occurrence of restoration that were recorded it comes out that:

– SCs located in sites categorized with Dsc/Dfc köppen climate classes as ID 10, ID 13, ID 17, ID 19 although subjected to recent maintenance works, showed low F-T risk on OGFSs;
– SCs located in Dfc or ET köppen climate classes as the ID 20 and the ID 23 showed higher risk thus asking for a more demanding maintenance in the future. This is also exacerbated by risk of floods and landslides as reported by Bertolin and Sesana [18].

## 4 Conclusions

In this Paper, a novel algorithm for estimating the structural integrity of the off-ground foundation stones of the whole group of 28 still existing Stave Churches has been proposed. The algorithm uses a single climatic variable as input i.e., the continuous daily time series of the average surface temperatures (ST) over a 0.25° x 0.25° area containing a stave church to reconstruct the last 70-years period (i.e., 1950-2020, Fig. 1) in ST variability. The analysis of the mean winter ST in 1951 and 2020, reported as maps, clearly highlights the impact of climate change over Norway.

The calculation of the temperature anomalies referring to the average daily ST respect two reference 30-years periods (i.e., FP:1961-1990 and RP:1991-2020) evaluates this climate change impact (Fig. 5) that does not manifest itself as an overall temperature increase in the average trend (30-day moving average), but rather through surface temperature dispersion and frequency modifications.

The FFT allows to analytically estimate such modifications (Fig. 6). The increase in heat loss and the variation of the periodicities have a double climatic effect: an increase in the number of F-T cycles in the winter-spring and autumn-winter transition periods, and a decrease in the duration of winters (gray area Fig. 6). Both these two effects may impact the structural integrity of OGFSs in the SCs.

On such premises, the main goal of this paper is the development of a damage function capable to assess the effect of such modifications on the durability (LT (t) function) of the stave churches foundation stones. For this purpose, three reference churches at high-, medium-, and low-risk have been selected (Figure 6, Figure 7) looking at the output of the algorithm implemented to build the function. Fig. 8 reports the parameters used by the LT (t) function (Equation (9)) i.e., the angular coefficients of the SD_3DD_ and SD_7DD_ linear regressions as visible in Fig. 7. Simulations of LT (t) functions of the 10 churches most at risk are forecasted over a time horizon of 200 years in Fig. 9 after the studies of the ST climatology. These functions offer an immediate graphical forecast of future required in situ surveys to verify the OGFS integrity, while equations (9), (10), (11) and (12) provide the same results analytically (Table 4). In conclusion, we have presented a new, simple, and immediate algorithm accompanied by a function capable of monitoring, comparing, and estimating the potential of frost damage on OGFSs of a historically and architecturally homogeneous group of heritage buildings.

The whole algorithm uses a single climate input parameter (i.e., the ST), making it easier to broadly exploit it through different sites comparison. The obtained results are important as they offer an effective risk analysis tool which may guide stakeholders in the prioritization of the asset maintenance activities as budget and planned outage time is often limited for all maintenance work that need to be carried out. In case high damage potential is detected by the algorithm, in-depth in situ survey needs to be carried out to verify the level of frost weathering on the stone foundations and its possible repercussions on the SCs superstructure.

In future research, the impact of climate change can further be investigated by implementing higher spatial and temporal resolution analysis for those cases at high-risk, possibly adding other meteorological parameters to further improved the algorithm or to extend the effectiveness of the durability function through verification within situ monitoring campaigns devoted to various structural materials.

## Declarations

### Author contribution statement

Chiara Bertolin: Conceived and designed the experiments; Analyzed and interpreted the data; Wrote the paper. Stefano Cavazzani: Analyzed and interpreted the data; Wrote the paper.

### Funding statement

Prof Chiara Bertolin was supported by Norges Forskningsråd [274749].

### Data availability statement

Data will be made available on request.

### Declaration of interests statement

The authors declare no conflict of interest.

### Additional information

No additional information is available for this paper.

## Footnotes

1 Reports on the SCPP, in Norwegian, available at: https://ra.brage.unit.no/ra-xmlui/handle/11250/176302?locale-attribute=en.
